# Effects of the Wall Temperature on Rarefied Gas Flows and Heat Transfer in a Micro-Nozzle

**DOI:** 10.3390/mi15010022

**Published:** 2023-12-22

**Authors:** Shurui Zhang, Yong Li, Xudong Wang, Songcai Lu, Yusong Yu, Jun Yang

**Affiliations:** 1 Hydrogen Energy and Space Propulsion Laboratory (HESPL), School of Mechanical, Electronic and Control Engineering, Beijing Jiaotong University, Beijing 100044, China; 21121387@bjtu.edu.cn; 2 Beijing Institute of Control Engineering, Beijing 100190, China; kyffwd@163.com (X.W.); 14151020@buaa.edu.cn (S.L.); 3 Changcheng Institute of Metrology and Measurement, Beijing 100095, China; yangjun@cimm.com.cn

**Keywords:** cold gas micro-nozzle, rarefied flow, DSMC method, needle valve opening ratio, large length-to-diameter ratio, micro-channel

## Abstract

When the satellite is in orbit, the thruster will experience drastic temperature changes (100–1000 K) under solar radiation, which will affect the rarefied gas flow state in the micro-nozzle structure of the cold gas micro-thruster. In this study, the effect of different wall temperatures on the rarefied flow and heat transfer in the micro-nozzle is investigated based on the DSMC method. The micro-nozzle structure in this paper has a micro-channel with a large length-to-diameter ratio of 10 and a micro-scale needle valve displacement (maximum needle valve displacement up to 4 μm). This leads to more pronounced multiscale flow characteristics in the micro-nozzle, which is more influenced by the change in wall temperature. At wall temperatures ranging from 100 K to 1000 K, the spatial distribution of local Kn distribution, slip velocity distribution, temperature, and wall heat flux distribution in the micro-nozzle were calculated. The slip flow region is located in the flow channel and transforms into transition flow as the slip velocity reaches approximately 50 m/s. The spatial distribution of the flow pattern is dominated by the wall temperature at small needle valve opening ratios. The higher the wall temperature, the smaller the temperature drop ratio in the low-temperature region inside the micro-nozzle. The results of the study provide a reference for the design of temperature control of micro-nozzles in cold gas micro-thrusters.

## 1. Introduction

In recent years, many major projects such as the “high-precision gravity field measurement”, “Tianqin Plan”, and “Taiji Plan” for gravitational wave detection, and the “Sound Seeking Plan” for the exploration of extraterrestrial civilization have been launched in succession [1,2]. The Micro Newton Thruster is the key to realizing “drag-free control” in the above space exploration missions and guaranteeing the technical requirements of the payload for the ultra-static working environment [3]. The current domestic and foreign Micro Newton Thrusters applied to the technology include cold gas thrusters, electrospray thrusters, RF ion thrusters, and cusped field thrusters [4,5,6], where the cold gas thruster technology has the advantages of high specific impulse, small mass, low power consumption, etc., and have been applied in spacecrafts.

Gas flow in a cold gas micro thruster operating under vacuum conditions is likely to be in continuum, slip, transition, and free molecular flow states before the gas is ejected from the nozzle into a vacuum environment [7]. Due to the difficulty of experimental operation caused by the micro-scale [8], numerical simulation is an effective alternative to exploring this phenomenon of rarefied gas flow. However, when the gas flow is in the free-molecular flow regime, the frequency of collisions between gas molecules is roughly comparable to the frequency of collisions between gas molecules and the surface of the solid object. Therefore, the continuous medium assumption, which presupposes the dominance of collisions between gas molecules, cannot be satisfied, nor can dynamic theory be applied to describe the behavior of rarefied gas. A more accurate gas kinetic theory is needed [9]. In this paper, the DSMC [10] approach is used to describe the behavior of rarefied gas flow inside a large length-to-diameter ratio micro-nozzle in a cold gas thruster system. The DSMC approach starts from a microscopic perspective, using a small number of simulated molecules to represent real fluid molecules. It simulates and calculates the movement and collisions of molecules via the probabilistic simulation method of statistical physics, which eventually results in a more accurate macroscopic physical process. Compared to macroscale nozzles, the rarefied gas flow inside micro-nozzles is more complex, and a very distinctive feature is the rarefaction effect [11,12,13]. According to the Knudsen number, the flow is classified into the following four types of regimes: continuum flow (Kn ≤ 0.01), slip flow (0.01 < Kn ≤ 0.1), transition flow (0.1 < Kn < 10), and free molecular flow (Kn ≥ 10) [14]. One of the main differences between slip flow and continuum flow is the occurrence of velocity slip and temperature jump conditions, which means the differences between the adjacent gas flow’s velocity and temperature with those of the wall [15]. Furthermore, according to general experience from macroscale flow studies, the effect of roughness on the friction coefficient is negligible when the relative roughness of the wall surface is less than 5% [16]. However, it has been shown that the wall roughness within the micro-channel still has an essential effect on the rarefied flow and heat transfer, even if it is small [8]. Kandlikar et al. [17] used an experimental method to measure the micro-channel flow and heat transfer processes at a relative roughness of 3.55% and found that the effect of roughness on the friction and heat transfer coefficients was significant. Shams et al. [18] used the NS equations coupled with first-order velocity slip boundary conditions to study the rarefied flow process in a micro-channel with rough walls (relative roughness 5%, Kn = 0.01, Ma = 0.4). The results show that gas compressibility and wall roughness change the pressure gradient distribution, reduce the mass flow rate, and increase the flow resistance.

**Figure 1:**
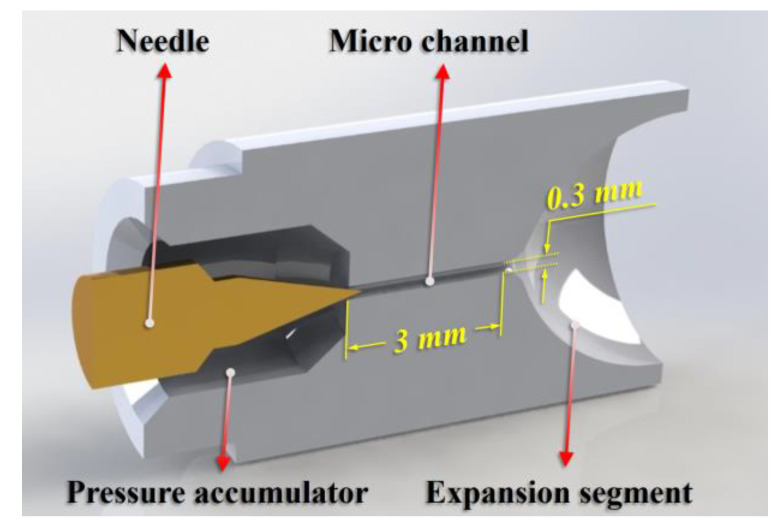
The micro-nozzle with a large length-to-diameter ratio micro-nozzle in the present study.

When a satellite is in orbit, the thrusters experience drastic temperature changes under the influence of solar radiation, which affects the energy transfer between the micro-nozzle and the internal fluid. Therefore, the effect of temperature on the gas flow state inside the micro-nozzle was studied. Alexeenko et al. [19] simulated the time variation characteristics of gas temperature and flow and the operating time limit of the thrusters using two-dimensional and three-dimensional micro-nozzle models. The results showed that the thrust and mass flow coefficients decrease with increasing wall temperature. Louisos et al. [20] investigated the effect of isothermal wall conditions in planar micro-nozzles on their subsonic layer growth. Their simulations showed that the heat loss in the flow behavior reduces the viscous effect and the size of the corresponding subsonic boundary layer, thus improving the performance of the micro-nozzle. Hameed et al. [21] focused on the effect of using heated or cooled walls to control the flow characteristics of micro-nozzles. The study demonstrated that subsonic boundary layer thickness could be reduced, and viscous losses can be mitigated by wall cooling. Sukesan et al. [22] studied numerical simulations of two-dimensional micro-nozzles with different wall temperatures for Kn > 0.001. They observed that the thrust and peak Mach number inside the nozzle decreases with increasing wall temperature, and the thickness of the exit subsonic layer increases with increasing wall temperature. Rafi et al. [23] simulated the gas flow state inside the micro-nozzle for different wall thermal conditions, and they found that the higher the wall temperature, the faster the flow expansion to the vacuum environment and the larger the diameter of the flow plume. The growth of the subsonic layer is attributed to the combined effects of the larger surface-to-volume ratio in micro-nozzles, the temperature dependence of fluid viscosity, and deceleration due to the Rayleigh-flow effect. However, there is a lack of research on micro-nozzle structures with a length-to-diameter of up to 10. The presence of a large length-to-diameter ratio micro-channels between the needle throat and the expansion section leads to more pronounced multiscale flow phenomena inside the micro-channel, which is more affected by the wall temperature. Figure 1 shows the micro-nozzle with a large length-to-diameter ratio micro-nozzle in the present study.

The purpose of this paper is to numerically study the effect of wall temperature on the flow patterns of rarefied gas inside the cold gas micro-nozzle with a large length-to-diameter of up to 10 based on the DSMC method and to reveal the effect of wall temperature on flow states, local Kn number, slip velocity, temperature, and wall heat flux distribution.

## 2. Numerical Simulation

### 2.1. Direct Simulation Monte Carlo Method

The Direct Simulation Monte Carlo method (DSMC) directly starts from the physical simulation of flow, which simulates the molecular motion, wall collision of rarefied gas flow, and the interaction between molecules. It has been proved that DSMC converges to the Boltzmann equation [24,25,26]. For low-speed rarefied flows in the near continuum regime, the discretized velocity method could offer greater efficiency and noise reduction compared to DSMC. However, DSMC has high efficiency in capturing the main features of rarefied flows and may be the most widely used approach for modeling rarefied gas flows [27]. And the simulation results are exactly consistent with the experimental results in terms of the overall effect and fine structure of the flow field. It uses a large number of simulated molecules to simulate real gas molecules. Therefore, compared with the molecular dynamics method (MD), it reduces a lot of computational complexity. The computer stores the position coordinates, velocity components, and internal energy of each simulated molecule. They change with time as the molecules move and collide with the boundary and with each other.

The main idea of the DSMC method is to decouple the motion and collision of molecules. Realizing correct collision sampling, that is, selecting appropriate collision pairs and realizing a certain number of collisions, is the key to matching collision and motion in ∆t time and making the simulation consistent with the real flow process. So far, different schemes for performing collision rates have been developed in the DSMC method [28]. This paper selects the No Time Counter (NTC) method [29], which is widely used in many simulations.

There are two core problems of the DSMC simulation method: molecular collision simulation and molecule–wall interaction simulation. The collision model between molecules includes the Hard Sphere (HS) model [30], the Variable Hard Sphere (VHS) model [31], the Variable Soft Sphere (VSS) model [32], the Generalized Hard Sphere (GHS) model [33], and the Generalized Soft Sphere (GSS) model [34]. Among them, the VHS is more widely used, and this model introduces two scattering angles θ and φ uniformly distributed in the spherical direction to describe the velocity direction of the particle sample pairs after the collision where the azimuthal angle φ is a uniform random number between 0 and 2π.

(1) $$\varphi =2\pi R_{f}$$

The elevation angle θ is a uniform random number between −1 and 1 and is solved by $R_{f}$:

(2) $$\cos\theta =2R_{f}-1$$

The relative velocity of the particle pairs after the collision is

(3) $$c_{r}^{*}=c_{r}^{*}[\left(\cos\theta \right)\hat{x}+\left(\sin\theta \cos\phi \right)\hat{y}+\left(\sin\theta \sin\phi \right)\hat{z}]$$

Thus, the velocities of the sample particles *i* and *j* after the collision are

(4) $$c_{i}^{*}=c_{cm}^{*}+\left(\frac{m_{j}}{m_{i}+m_{j}}\right)c_{r}^{*}$$

(5) $$c_{j}^{*}=c_{cm}^{*}-\left(\frac{m_{i}}{m_{i}+m_{j}}\right)c_{r}^{*}$$

where $c_{i}^{*}$ and $c_{j}^{*}$ are the velocities after the collision; $m_{i}$ and $m_{j}$ are the masses of the particle sample pair; $c_{cm}^{*}$ is the velocity of the particle sample pair at the center of mass after the collision; and $c_{r}^{*}$ is the relative velocities of the particle sample pair after the collision.

The collision model between molecule and wall includes specular reflection, complete diffuse reflection, and their combinations of Maxwell-type reflection, as well as the CLL [35] object–face reflection model, which is closer to the physical reality. The CLL model assumes that the normal and tangential velocities in the molecular reflection process are independent of each other and introduces a dispersive kernel function to describe the normal and tangential velocity probabilities of the reflected molecules. The dispersive kernel function determines the relationship between incident molecules and reflected molecules. The dispersion kernel function of tangential velocity and normal component is expressed as follows:

(6) $$R\left(v_{i},v_{r}\right)=\frac{1}{\sqrt{\pi \sigma_{t}\left(2-\sigma_{t}\right)}}\exp\left[-\frac{{\left(v_{r}-\left(1-\sigma_{t}\right)v_{i}\right)}^{2}}{\sigma_{t}\left(2-\sigma_{t}\right)}\right]$$

(7) $$R\left(u_{i},u_{r}\right)=\frac{2u_{r}}{\alpha_{n}}I_{0}\exp\left[-\frac{u_{r}^{2}+\left(1-\alpha_{n}\right)u_{i}^{2}}{\alpha_{n}}\right]\cdot \left[\frac{2{\left(1-\alpha_{n}\right)}^{1/2}u_{r}u_{i}}{\alpha_{n}}\right]$$

where $v_{i}$ and $v_{r}$ are the tangential velocities of incident molecules and reflective molecules; $u_{i}$ and $u_{r}$ are the tangential velocities of incident molecules and reflective molecules; and $\sigma_{t}$ and $\alpha_{n}$ are tangential momentum adjustment coefficient and normal energy adjustment coefficient, which are set to 0.8 and 1.0, respectively.

### 2.2. Simulation Models

**Figure 2:**
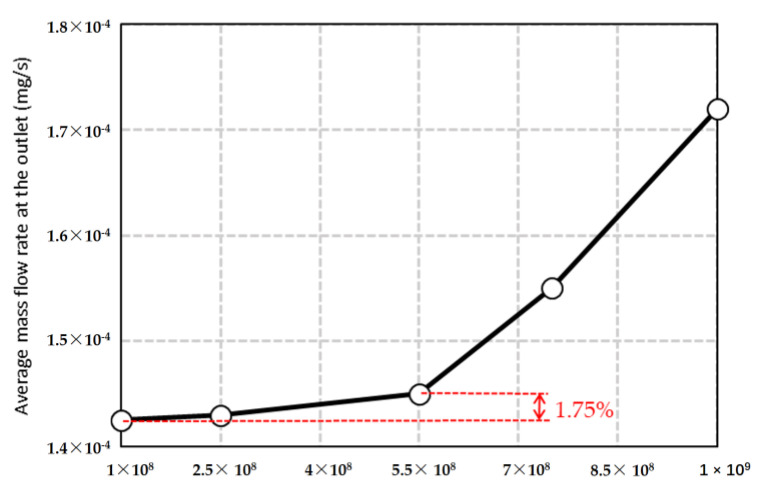
Effect of equivalent molecular number of nitrogen molecular sample package.

For the inlet boundary, we set the molecular number density to $1.9011\times 10^{25}\times \left(1+0.1sin\left(2\pi \times 10^{6}t\right)\right)\;1/m^{3}$ to correspond to the actual inlet pressure of 0.15 MPa and the pressure fluctuations of the incoming upstream flow. Due to the existence of a thermal control system in the high-pressure inlet region of the actual micro-nozzle, the inlet gas temperature was stable at 293.15 K. The nitrogen molecular mass was 46.5 × 10^−27^ kg/m^3^, and the equivalent diameter was 3.17 × 10^−10^ m. Since the actual outlet is a vacuum environment, the molecular number density at the outlet boundary was set to zero. Figure 2 shows the effect of the equivalent molecular number of the nitrogen molecular sample package. According to the comparison results of the average mass flow rate at the outlet, the equivalent molecular number of the nitrogen molecule sample package is 5.5 × 10^8^, which has a high calculation accuracy. The relative deviation of the calculation results caused by continuing to reduce the equivalent molecular number is 1.75%. At the same time, it has a relatively large number of equivalent molecules, which can reduce the number of nitrogen molecular sample packages, thus reducing the calculation time consumption.

A fixed calculation time step with a value of 1 × 10^−9^ s is used, which should be less than the mean time of the collision. To verify the accuracy of the selected time step, we compared the time step to the ratio Δx/(3 × u0), where Δx is the applied maximum cell size and u0 is the most probable velocity. The results show that the calculated adaptive time step is always greater than the set fixed time step, so it meets the requirements. The rarefied gas flow and heat transfer in the micro-nozzle reach a quasi-stable state at 0.1 ms.

**Figure 3:**
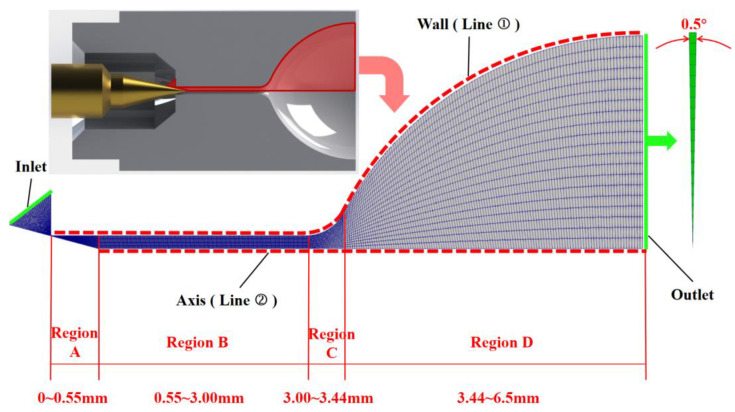
Computational domain and mesh division.

The micro-nozzle structure, as shown in Figure 3, includes a high-pressure section, a large length-to-diameter ratio micro-channel, and a nozzle expansion section, where (1) the needle valve cone angle is 30°, (2) the micro-channel inner diameter is 0.3 mm, the length is 3 mm, that is, the aspect ratio is 10; (3) the expansion section outlet diameter is 5 mm; and (4) as the displacement of the needle valve increases from 0 μm to 4 μm, the needle valve opening is defined as 0% to 100%. The micro-nozzle calculation domain is shown in Figure 3. Due to the high-pressure section being a dense continuum flow, to reduce the calculation consumption, only a small part of the high-pressure section is included in the present computational domain. The structured mesh of the computational domain is adopted by the OpenFOAM’s BlockMesh application, and the wedge-shaped computational domain with a circumference of 0.5° is filled with hexahedral cells. The micro-nozzle is divided into four parts according to the structure as follows: Region A: needle valve throat; Region B: micro-channel; Region C: micro-nozzle expansion Section 1; and Region D: micro-nozzle expansion Section 2.

**Figure 4:**
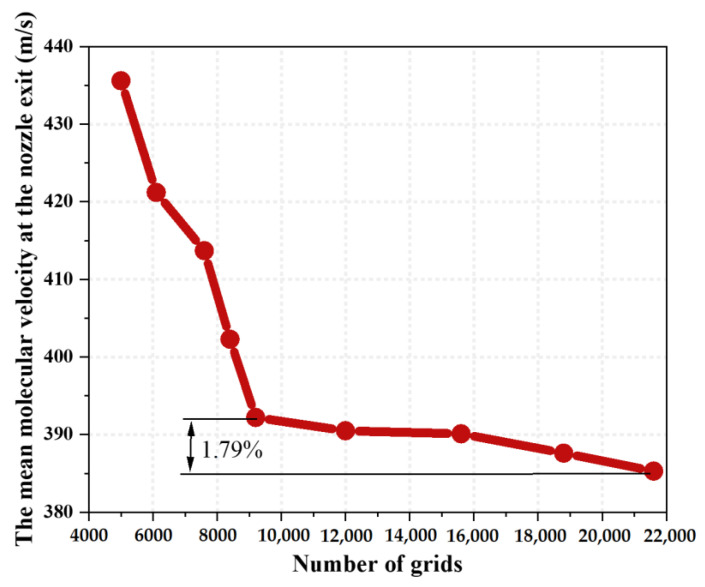
Effects of the grid number on the mean molecular velocity at the nozzle exit face.

The total number of meshes in the computational domain is 9200. According to the statistics of the mean molecular velocity at the outlet face in Figure 4, this total number of meshes is small and has high accuracy. The deviation is less than 2% compared to the total number of refined meshes of 21,600, and the influence of continuing to increase the number of meshes on the computational results is small.

In order to smooth the DSMC data in the predicted results, particularly slip velocity, a filtering post-processor is utilized. In this method, the sampled macroscopic properties (*F*) are averaged over four neighboring cells, as given below [36].

(8) $$F_{i,j}=0.2\left(F_{i-1,j-1}+F_{i-1,j}+F_{i,j}+F_{i,j+1}+F_{i+1,j+1}\right)$$

### 2.3. Validations of the Numerical Results

**Figure 5:**
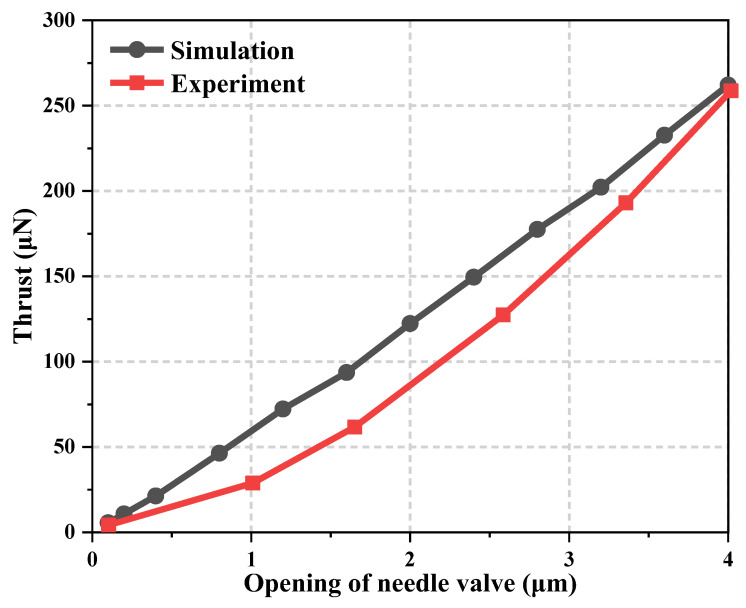
Comparison of experimental and simulation thrust forces under different valve opening ratios.

To verify the accuracy of the mathematical models, the thrust force data of the different needle valve opening ratios are obtained by simulation and compared with the experimental results under the same conditions. The indirect force measurement system based on the torsion pendulum platform was adopted, which is consistent with the experimental platform of Liu et al. [37]. The measurement system consists of the vacuum chamber, damper, pendulum, counterweight, displacement device, micro-thruster, and calibration system. A high-precision capacitive displacement meter was used to measure arm displacement. As shown in Figure 5, the thrust force varies with the different needle valve opening ratios in the same trend, and the error is within the acceptable range.

## 3. Results and Discussion

### 3.1. Flow Regime Spatial Distributions and Local Kn Distribution

**Figure 6:**
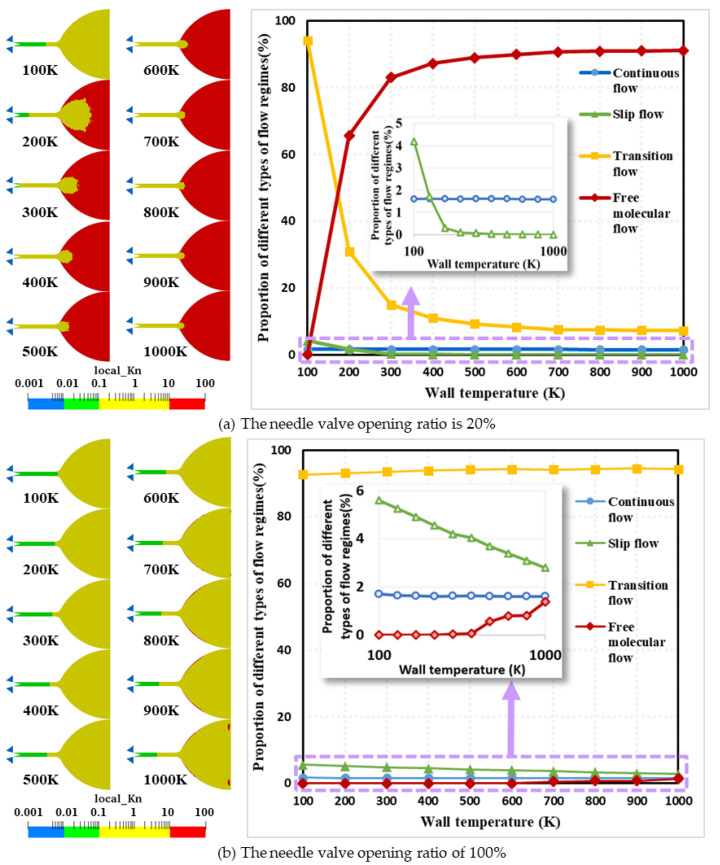
Flow regime spatial distributions and area share curve of each flow region at different wall temperatures and needle valve opening ratios at 1 ms. (**a**) The needle valve opening ratio at 20%. (**b**) The needle valve opening ratio at 100%. Left side: spatial distribution of local Kn number. Four colors are used to mark the different flow regimes. The blue area represents the continuum flow regime with no-slip condition. The green area represents the continuum flow regime with slip conditions. The yellow and red regions represent the transition flow and free molecular flow, respectively. Right side: the area ratio of each flow regime.

The spatial distribution of the flow regime inside the micro-nozzle at different wall temperatures at 1 ms is given in Figure 6, where (a) is the result obtained under the condition of 20% needle valve opening and the needle valve opening ratio of (b) is 100%. The local Knudsen number is defined as Kn = λ/L, where L is expressed as the scale length of the macroscopic gradients, i.e., $L=\frac{\rho }{d\rho /dx}$. Different colors in the figure represent different flow regions. Blue indicates continuum flow region with Kn ≤ 0.01; green indicates slip flow region with 0.01 < Kn < 0.1; yellow indicates transition flow region with 0.1 < Kn < 10; and red indicates free molecular flow region with Kn ≥ 10. The results in Figure 6a show that the wall temperature has a great influence on the spatial distribution of the flow regimes inside the micro-nozzle under the condition of 20% needle valve opening, and as the wall temperature increases from 100 K to 1000 K, the flow regime in the expansion section transitions from the transition flow to the free molecular flow. Among them, the continuum flow region is located in the high-pressure area upstream of the throat. With the increase in wall temperature, the area of continuum flow has almost no change, which is always located in the vicinity of 1.6%. The slip flow region is distributed in the micro-channel, with the increase in wall temperature, its area ratio from 4.19% in the abrupt decrease to 0.09% at 400 K, as the wall temperature continues to rise to 1000 K, the area of slip flow region slowly decreases to 0.07%. Transition flow exhibits a tendency to move from the nozzle expansion section to the throat. With the increase in wall temperature, the transition flow region in the nozzle expansion section gradually shrinks, the transition flow in the micro-channel slowly advances in the direction of the upstream flow direction, and the overall area reduced rapidly from 94.17% at 100 K to 9.29% at 500 K, and then gradually decreases to 7.22% at 1000 K. The free molecular flow region from the outlet of the nozzle expansion section gradually expands in the direction of the micro-channel, from 0.03% at the wall of the expansion section at 100 K to 89.03% at 500 K, and then gradually increases to 91.19% at 1000 K. The reason for the above phenomenon is that the increase in wall temperature leads to the expansion of the gas inside the micro-nozzle, the mean molecular free path gradually increases and leads to an increase in the Kn number.

The results in Figure 6b show that the wall temperature has a small effect on the spatial distribution of the flow regimes inside the micro-nozzle under 100% needle valve opening conditions, and as the wall temperature increases from 100 K to 1000 K, the expansion section is always filled with a large amount of transition flow. Among them, continuum flow in the high-pressure area mainly exists upstream of the throat. There is also a small amount of continuum flow downstream of the needle valve at 100 K. With the increase in wall temperature, the area of continuum flow in the downstream part gradually decreases, but the continuum flow between 100 K and 1000 K only decreases by 5.81%; the slip flow is located in the micro-channel; with the increase in the wall temperature, its area ratio from 5.60% approximately linear declines from 5.60% to 2.79%; and the area of the transition flow increases linearly from 92.69% at 100 K to 94.26% at 600 K. Then, the area of the transition flow fluctuates due to the growth of the free molecular flow and the decay of the slip flow; the free molecular flow appears only at 500 K and is distributed only at the wall of the nozzle expansion section and the nozzle exit. With the increase in wall temperature to 1000 K, only 1.38% of the free molecular flow also appears. The reason for this phenomenon is the higher temperature of the gas near the wall, resulting in a larger mean molecular free path than the molecules in the exit plane, i.e., the generation of free molecular flow.

Comparing the flow regime spatial distributions at different needle valve openings, it can be found that the wall temperature at the valve opening ratio of 20% has a more significant effect, while the flow state at the valve opening ratio of 100% barely changes. The free molecular flow area at 100% needle valve opening is only 89.81% of that at the 20% condition. The reason for this phenomenon is that the molecules passing through the throttle area of the valve are smaller at smaller openings, resulting in more sparse molecules in the micro-channel.

**Figure 7:**
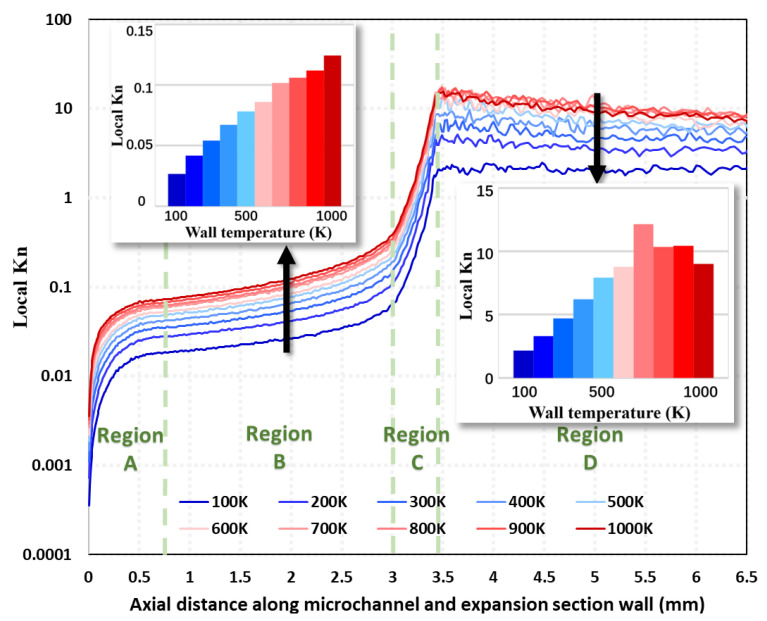
Local Kn distribution along the micro-channel and nozzle expansion section near the wall (Line ➀ in Figure 3) at different wall temperatures under 100% needle valve opening ratio at 1 ms (The inset means a histogram of the Local Kn number at different wall temperatures).

The flow regime partition map in Figure 6 can only provide qualitative information on the flow region at a macroscopic level. The results of the local Kn distribution along the inner wall of the micro-channel and the expansion section (i.e., Line ➀ Figure 3) in Region A, B, C, and D (See Figure 3) at different wall temperatures at 100% needle valve opening are given in Figure 7. In Region A, the local Kn number increases rapidly from 10^−4^ to 10^−2^ along the axial downstream direction. As the temperature increases, the local Kn number in the same position increases. Increasing the wall temperature decreases the local gas density, which leads to an increase in the mean molecular free path. When the nitrogen gas enters Region B, the local Kn number growth rate becomes significantly slower due to the wall constraint of the micro-channel. When the gas flows into Region C, the local Kn number increases significantly again. The peak value of local Kn reaches 10^2^. The geometric expansion structure of Region C leading to gas expansion is responsible for the above results. However, when the gas flows through Region D, the local Kn number appears to remain constant (i.e., wall temperature is 100 K) or even decrease (i.e., wall temperature larger than 100 K). The radial length of the nozzle expansion section of the region gradually reduces the growth rate. The nitrogen molecules in the expanded section of the nozzle cannot expand sufficiently, limiting the increase in the mean molecular free path.

**Figure 8:**
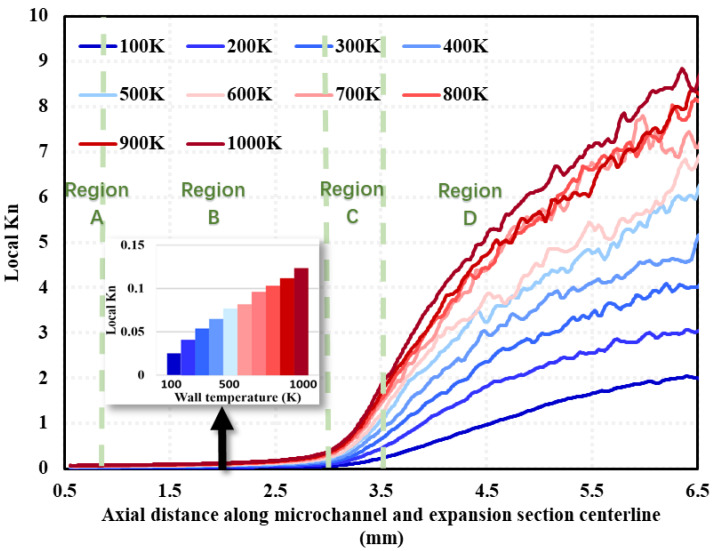
Local Kn distribution along the micro-channel and nozzle expansion section near the centerline (line ➁ in Figure 3) at different wall temperatures under 100% needle valve opening ratio at 1 ms (The inset means a histogram of the Local Kn number at different wall temperatures at 2 mm).

The results of the local Kn distribution along the central axis of the micro-channel and the expansion section (i.e., Line ➁ in Figure 3) in Region A, B, C, and D (See Figure 3) at different wall temperatures at 100% needle valve opening are given in Figure 8. The results show that three different regions of the curve appear: (1) in Region B, the local Kn along the mid-axis of the micro-channel shows a slow growth trend, and the growth rate increases with the increase in wall temperature; (2) in Region C, the local Kn along the mid-axis of the nozzle expansion Section 1 gradually grows, and the growth rate gradually becomes more significant, which is due to the geometric expansion structure of Region C; and (3) when the gas flows into Region D, the local Kn along the axis of the nozzle expansion Section 2 keeps growing, but the growth rate gradually becomes smaller, which is due to the limit of the nozzle structure.

### 3.2. Mach Number and Slip Velocity Distribution

**Figure 9:**
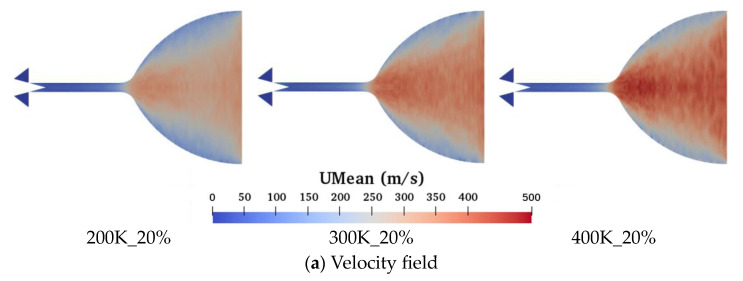

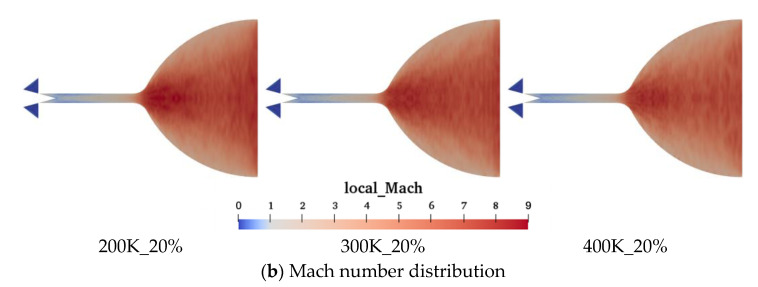
Time-averaged velocity field and Mach number distribution of axial symmetry surface at different wall temperatures and the needle valve opening ratio of 20% at 1 ms.

Figure 9 shows the distribution of the time-averaged velocity field and Mach number calculated up to 1 ms for wall temperatures of 200 K, 300 K, and 400 K at 20% needle valve opening ratio conditions. The Mach number is defined as $Ma=U/\sqrt{\gamma RT}$, where U, γ, R, and T represent the free-stream velocity, ratio of specific heat, gas constant, and temperature, respectively. The results show that the acceleration of nitrogen molecules occurs near the outlet of the microchannel. The velocity of the nitrogen molecules increases as the wall temperature increases. In contrast, the Mach number decreases gradually, which is due to the greater effect of temperature on the Mach number.

**Figure 10:**
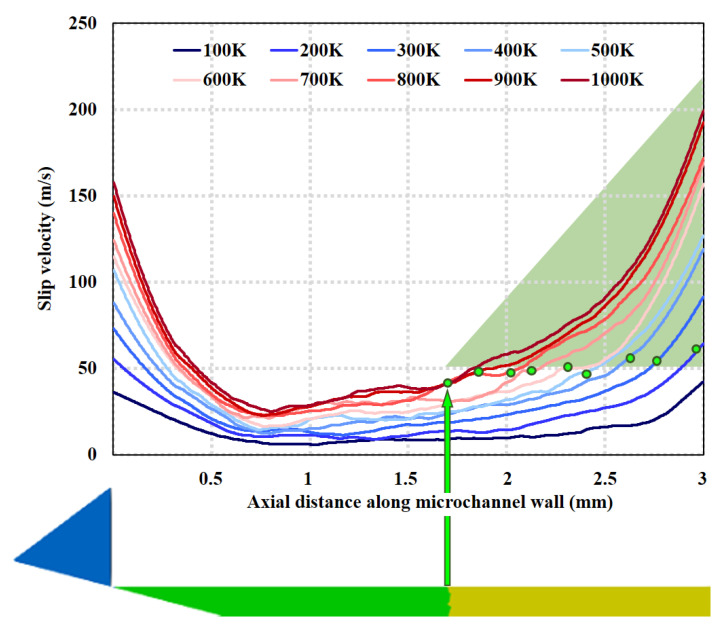
Slip velocity distribution along the micro-channel near the wall (Line ➀ in Figure 3) at different wall temperatures under 100% needle valve opening ratio at 1 ms.

There is a significant velocity difference between the boundary surface of the micro-channel and the adjacent fluid, which is known as the slip velocity [23]. Figure 10 shows the distribution of slip velocity near the wall surface of the micro-channel at different wall temperatures at 100% needle valve opening. The results in the figure show that the slip velocity increases with temperature, which is the same as the results of Rafi et al. [23]. In addition, along the wall surface of the micro-channel, the slip velocity first decreases in Region A and then increases slowly to near 50 m/s before accelerating. This is due to the fact that Region A is in the dilated section downstream of the needle valve, where the velocity gradually decreases because it is currently in subsonic flow. While the slow-growing region is in the slip flow state, the accelerated-growing region is in the transition flow state. Record the location information of the intersection of slip flow and transition flow at different temperatures in Figure 6b (i.e., the intersection of green and yellow areas in the figure) on the corresponding temperature curve in Figure 10. The results show that the slip velocity at the intersection is approximately concentrated near 50 m/s. The green shaded triangle means that the part with a slip velocity greater than 50 m/s is a continuous flow. By comparison, it can be seen that the intersection interface of slip flow and transition flow judged by local Kn is the same as the intersection interface judged by slip velocity.

### 3.3. Temperature and Wall Heat Flux Distribution

**Figure 11:**
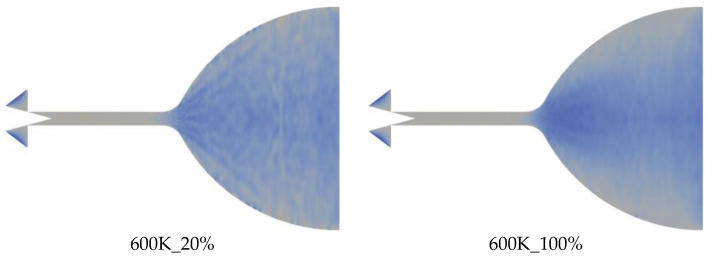

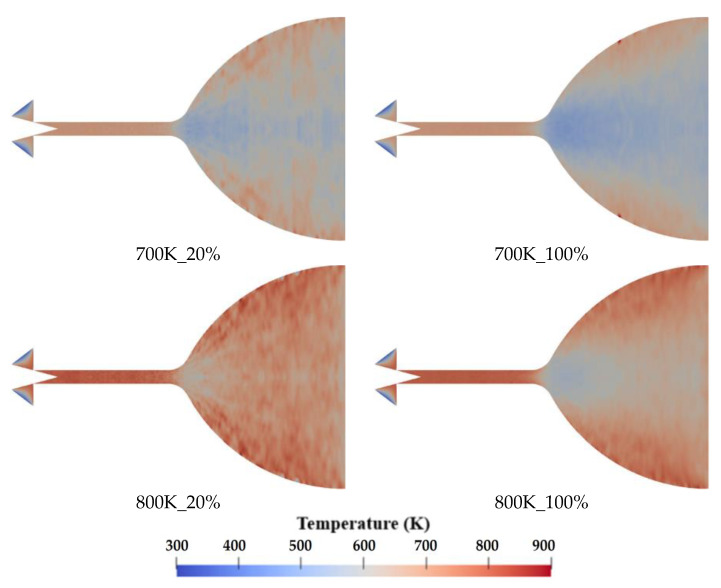
Time-averaged temperature field of axial symmetry surface at different wall temperatures and different needle valve opening ratios at 1 ms.

Figure 11 shows the distribution of the time-averaged temperature field calculated up to 1 ms for wall temperatures of 600 K, 700 K, and 800 K at 20% and 100% needle valve opening ratio conditions, respectively. The temperature is defined as $T=\frac{2}{3}F_{N}\cdot \left(\frac{1}{2}\bar{m}{\bar{c}}^{2}\right)/\left(\bar{N}\cdot k_{B}\right)$, where $F_{N}$ is the equivalent molecular number of nitrogen molecular sample package; $\bar{m}$ and $\bar{c}$ are the total mass of gas molecules within the cell and the mean of their migration velocity; $\bar{N}$ is the time-averaged number of DSMC particles within a cell; and $k_{B}$ is the Boltzmann constant (1.380649 × 10^−23^ J/K). The results show that the low-temperature region is distributed near the mid-axis of the nozzle expansion section, and the temperature is lower near the micro-channel section. This is due to the acceleration of gas molecules, which reduces the internal energy in the center of the nozzle expansion section. Compared with the time-averaged temperature field distribution at 100% opening, the temperature field distribution at 20% opening is more uniform, and the low-temperature region near the central axis is not apparent.

**Figure 12:**
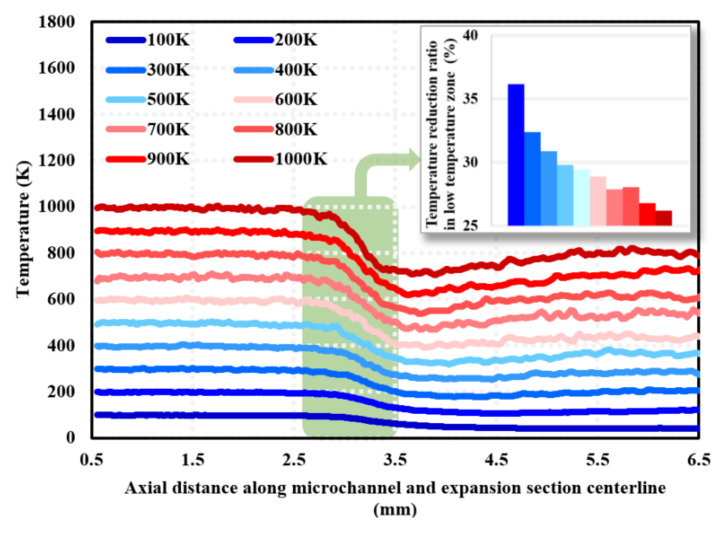
Temperature distribution along the centerline of the micro-channel and nozzle expansion section (i.e., Line ➁ in Figure 3) at different wall temperatures at 1 ms.

Figure 12 gives the temperature distribution along the mid-axis of the micro-channel and expansion section (i.e., Line ➁ in Figure 3) for different wall temperatures at a 100% needle valve opening ratio. The results show a significantly low-temperature zone in Region C, which is consistent with the results in Figure 11. The temperature reduction rate in the low-temperature region is 26.18–36.15%, and the magnitude of temperature reduction in the low-temperature region becomes smaller with the increase in wall temperature.

**Figure 13:**
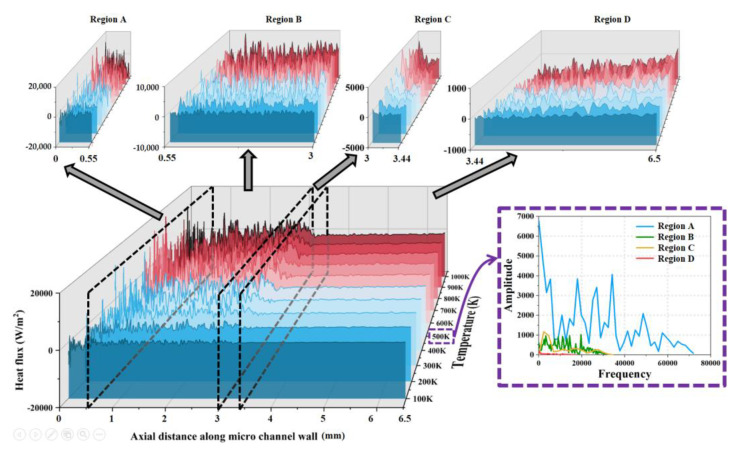
The distribution of heat flux along the wall (i.e., Line ➀ in Figure 3) at different wall temperatures at 1 ms and the spectrogram of heat flux at 500 K.

Figure 13 shows the distribution of heat flux along the wall at different wall temperatures, which is divided into four regions according to Figure 3. Via the fluctuation curve of the heat flux combined with the spectrum analysis at 500 K, the results show that the fluctuation range of the heat flux of these four regions gradually decreases. That is, the heat flux of Region A fluctuates between ±20,000 W/m^2^; the heat flux of Region B fluctuates between ±10,000 W/m^2^; the heat flux of Regions C fluctuates between ±5000 W/m^2^; and the heat flux of Region D fluctuates between ±1000 W/m^2^. In addition, the frequency of fluctuations in the heat flux of the wall of these four regions gradually decreases. The above phenomenon is due to the gradual increase in the radial size of these four sections, the number of nitrogen molecules impacting the wall drops, and the energy exchange decreases, so the fluctuation range and frequency of heat flux are reduced.

**Figure 14:**
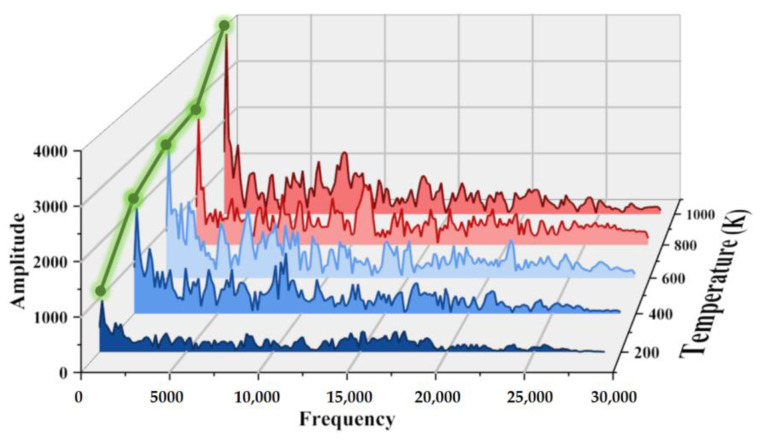
Spectrum diagram of heat flux along the wall (i.e., Line ➀ in Figure 3) at 1 ms for different wall temperatures.

Figure 14 shows the spectrum diagram of the heat flux at wall temperatures of 200 K, 400 K, 600 K, 800 K, and 1000 K. The results show that as the wall temperature increases, the amplitude of the heat flux increases. This is because as the temperature difference between the wall and the nitrogen increases, more heat is transferred when molecules collide with the wall, which is expressed as an increase in the heat flux along the wall. In addition, the increase in nitrogen temperature leads to a more violent thermal movement of the nitrogen molecules, which promotes the impact and heat exchange with the walls.

## 4. Conclusions

In this paper, the DSMC method is used to calculate the rarefied gas flow in a micro-nozzle with a large length-to-diameter ratio structure inside a cold gas micro thruster, and the effect of wall temperature on the flow characteristics is investigated. The flow states, local Kn number, slip velocity, temperature, and wall heat flux distribution in the micro-nozzle are discussed. The main results are as follows.

(1) Free molecular flow is concentrated near the wall of the nozzle expansion section and the nozzle outlet. The area of the free molecular flow region grows gradually with the increase in wall temperature. In the case of small needle valve openings, the flow regime spatial distributions are more significantly affected by the wall temperature. The area of the free molecular flow region of the needle valve opening at 100% is only 89.81% of that at 20%.

(2) The intersection of slip flow and transition flow in the large length-to-diameter ratio micro-nozzle is concentrated in the micro-channel, and the slip velocity at the junction is approximately concentrated near 50 m/s. The intersection of slip flow and transition flow judged by the local Kn is the same as the intersection evaluated by slip velocity.

(3) There is a low-temperature region in the expansion part of the micro-nozzle, the sudden drop in fluid temperature due to the extensive conversion of molecular energy into kinetic energy. The rate of temperature reduction in the low-temperature region is in the range of 26.18–36.15% and becomes smaller with the increase in wall temperature.

(4) The distribution of heat flux along the wall is divided into four regions due to the different radial dimensions of the micro-nozzle. As the wall temperature increases, more heat will transfer when the molecules collide with the wall, and the amplitude of the heat flux along the wall will increase.

## Data Availability

Some data, models, or codes that support the findings of this study are available from the corresponding author upon reasonable request. The data are not publicly available due to restrictions privacy.
